# Synchronous occurrence of HPV-associated cervical squamous cell carcinoma (FIGO IIA) in prolapsed uterus and adenocarcinoma of the anal canal cT1N0M0: Case report

**DOI:** 10.1097/MD.0000000000028004

**Published:** 2021-12-17

**Authors:** Andrzej Skręt, Joanna Trawińska, Joanna Bielatowicz, Mariusz Książek, Beata Niewęgłowska-Guzik, Andrzej Radkowski, Jaromir Kargol, Joanna Skręt- Magierło, Edyta Barnaś, Bogusław Gawlik

**Affiliations:** a Department of Obstetrics and Gynecology with Oncological Gynecology, Health Care Center, Krakowska 91, Dębica, Poland; b Department of Obstetrics and Gynecology, Frederic Chopin Provincial Clinical Hospital No. 1, Fryderyka Szopena 2, Rzeszow, Rzeszow, Poland; c Clinical Department of Histopathology, St Hedwig the Queen Clinical Provincial Hospital No. 2 in Rzeszow, Lwowska 60, Rzeszow, Poland; d Department of Pathology, Nonpublic Health Care Centre, Korczyńska 31, Krosno, Poland; e Radiotherapy Unit with the Department of Radiotherapy, St Luke Provincial Hospital, Lwowska 178a, Tarnow, Poland; f Department of Radiology and Diagnostic Imaging, Clinical Hospital No1 in Rzeszow, Rzeszow, Poland; g Institute of Medical Sciences, Medical College of Rzeszow University, Rejtana 16c, Rzeszow, Poland; h Institute of Health Sciences, Medical College of Rzeszow University, Rejtana 16c, Rzeszow, Poland.

**Keywords:** anal canal adenocarcinoma, cervical cancer in prolapsed uterus, synchronous anogenital cancers

## Abstract

**Rationale:**

Guidelines of rare synchronous tumours treatment are often unavailable due to lack of wide prospective studies. Additionally, their management is not just a simple sum of coexisting tumours management and has to regard many circumstances like symptoms, age, comorbidities, advancement.

**Patient concerns:**

Herein, we report a case of an 81-year-old woman who presented with bleeding from the prolapsed uterus.

**Diagnoses:**

Based on physical examination, that is, speculum examination, bimanual, and per rectum, followed by rectoscopy and histopathology, the diagnosis of cervical squamous cell carcinoma FIGO IIA2 in prolapsed uterus with anal canal adenocarcinoma cT1N0M0 was made.

**Interventions:**

Dominating complaint of bleeding from prolapsed cervix was managed with radical vaginal hysterectomy in conjunction with wide colpectomy preceded by laparoscopic pelvic and paraaortic lymphadenectomy. Due to the lack of consent for removal of the anus, only radiotherapy was applied instead.

**Outcomes:**

The patient underwent magnetic resonance image follow-up. No recurrence was found at 18 months.

**Lessons:**

Imaging is useful method of synchronous cancers diagnostics. These cancers may vary in aetiology and stage. Cervical cancer may be co-existing with another anogenital cancer. Therapy of synchronous cancers should be individualized taking into account patient's consent, age, physical condition, and comorbidities.

## 1 Introduction

Multiple primary tumours (MPT) are diagnosed when more than 1 tumour in the same or a different organ appears in 1 patient. The frequency of MPT ranges from 1% to 16% depending on primary localisation.^[1]^ Among MPT, 2 entities of tumours: synchronous and metachronous are identified. The synchronous one occurs when the second primary tumour is diagnosed in less than 6 months of the primary cancer. In case this period is more than 6 months, they are referred to as metachronous tumours.^[1]^

One tumour increases the risk of another. Standardized incidence ratio (SIR) defines how many times the risk of another cancer is increased after the first neoplasm occurrence. Some etiological factors are responsible for this phenomenon and they include, although not exclusively, human papilloma virus (HPV) infections. In HPV-related cancer patients pooled SIRs for second primary cancers amounts to 1.75 (95% confidence interval 0.66–4.67).^[2]^ According the Cancer Statistic, which estimated new cancer occurrence after primary one, patients suffering from cervical cancer (CC) have an increased risk of anal canal cancer (ACC), whereas those suffering from ACC have increased risk of CC.^[3]^ Despite the above data, studies on the coexistence of these neoplasms are rare. According to the authors of this study, there is only 1 report on the incidence of the synchronous CC in a patient with the ACC.^[1]^ The latter author emphasizes the difficulties in diagnosis and the lack of generally accepted guidelines for these synchronous neoplasms.

An additional factor that hinders treatment is the coexistence of cancer with other pathologies. Pelvic organ prolapse is a pathology that rarely coexists with CC. However, due to prolonged irritation of prolapsed organs, it may be involved in the etiopathogenesis of CC.^[4]^ Correction of this pathology may be a part of the surgical treatment of this tumour.^[5–9]^

## 2 Case report

**Figure 1:**
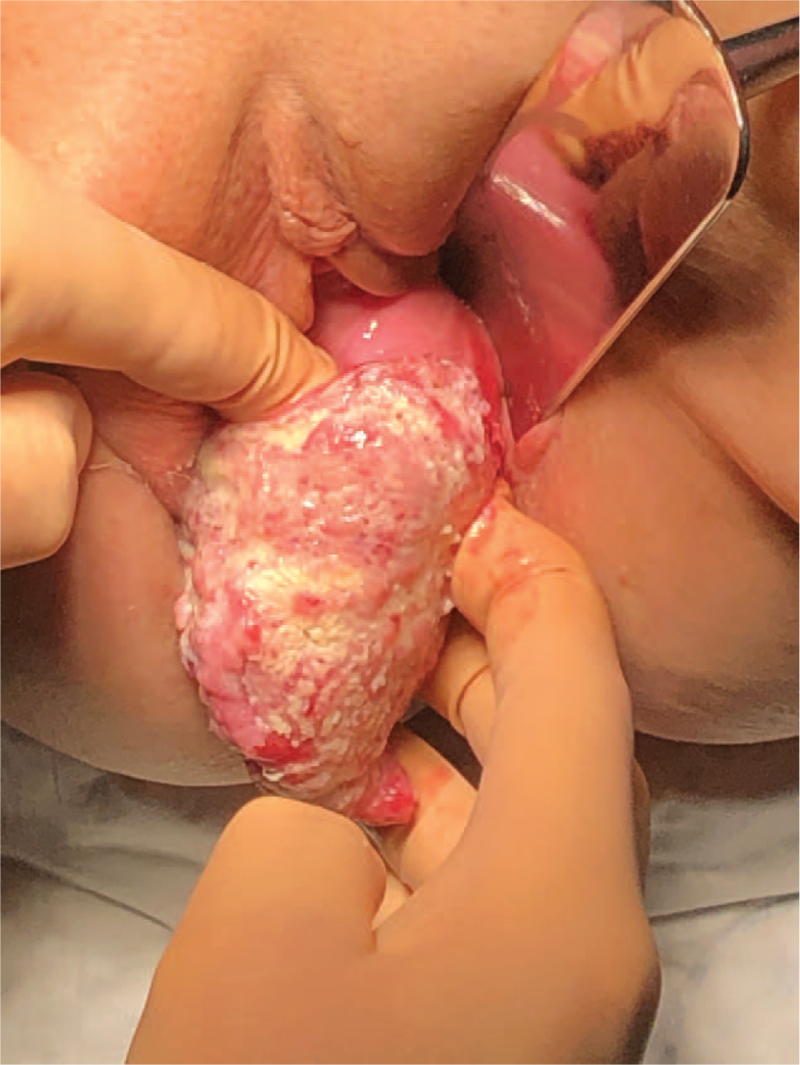
Cervical cancer in the prolapsed and elongated cervix.

An eighty year-old female presented with bleeding from the prolapsed uterus. The patient was admitted to the department of Gynaecology/Obstetrics and Gynaecological Oncology of Hospital in Dębica, Poland. In an obstetric history, she reported 3 vaginal births, the last one at the age of 30. Last menstruation was at the age of 52. She did not complain of any past diseases or current chronic diseases, she did not report any addictions and remained in 1 relationship. On admission, a large part of the vagina containing the cervix and a part of the uterine body was found below the vaginal introits (International Continence Society grade 3). Examination revealed an exophytic infiltrating lesions covering entire surface of hypertrophic cervix spreading to the vaginal fornices (Fig. 1).

Bimanual vaginal examination revealed a small myoma of 2 cm in diameter in the uterine body, adnexa impalpable. Per rectal examination showed a tumour in the anal canal. Moreover, the parametria were uninvolved in this examination.

**Figure 2:**
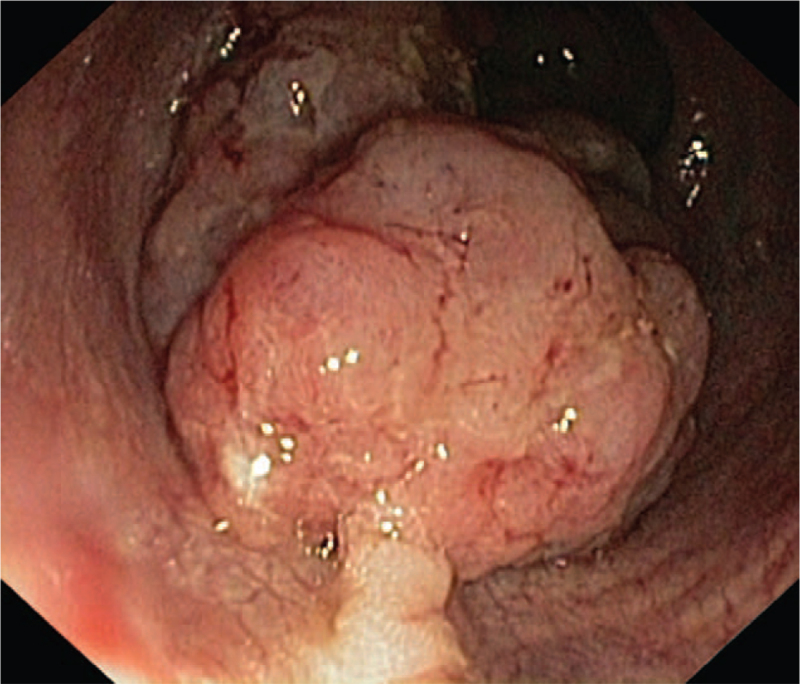
Rectoscopic view: in the foreground a visible polyp, in the background, an infiltrating lesion occupying the posterior and right walls of the anal canal.

**Figure 3:**
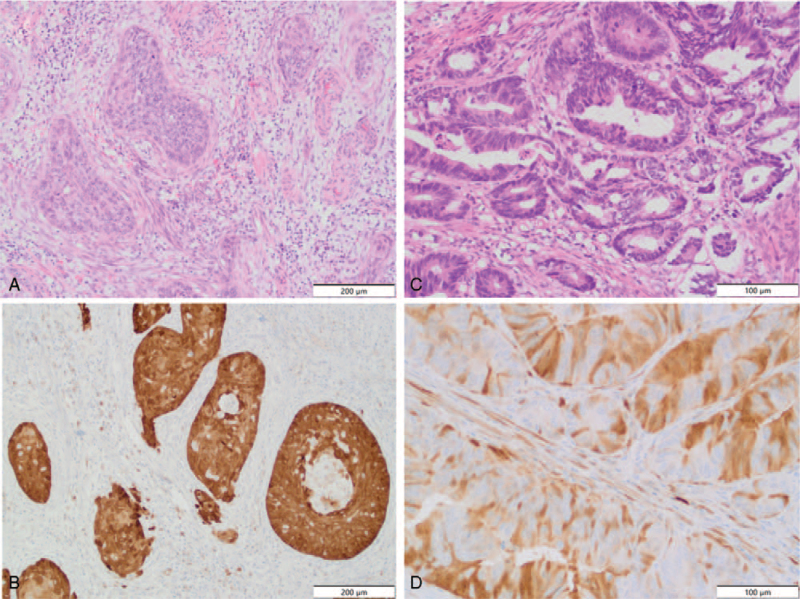
Histological images of the cervical and anal canal lesions. (A) Moderately differentiated squamous cell carcinoma of the cervix with marked desmoplastic stromal reaction. H&E stain, objective × 10. (B) Strong and diffuse p16 staining in cervical squamous cell carcinoma, objective × 20. (C) Moderately differentiated adenoracinoma of the rectum, H&E stain, objective × 20. (D) Areas of strong but in a mosaic fashion p16 staining in rectal adenocarcinoma, objective × 20.

In the diagnostic procedure, the lesion in the cervix was sampled parallelly with curettage of the cervical canal and the uterine cavity. Then, rectoscopy was performed with sampling of the anal canal lesion for histopathology. The rectoscopic image is shown in Figure 2 and the microscopic images of the cervical and anal canal samples in Figure 3.

A polypoid lesion in the rectoscopy measuring 2.5 cm in diameter just behind the external sphincter and an infiltrating lesion occupying the posterior wall of the rectum, 4 cm away from the anal rim were found (Fig. 2).

**Figure 4:**
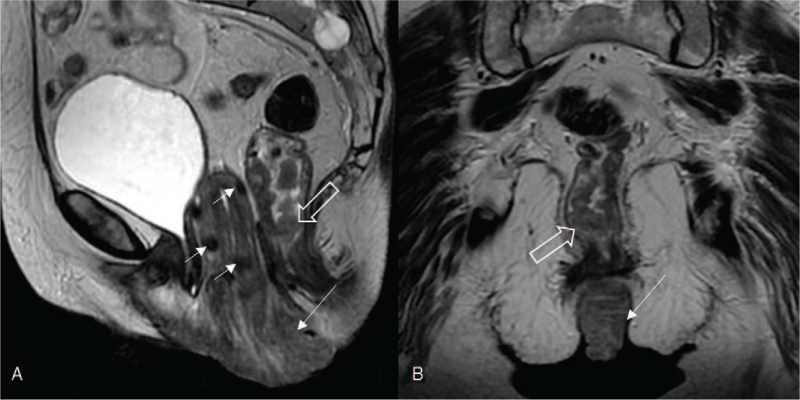
Sagittal (A) and coronal (B) T2-weighted magnetic resonance images. The prolapsed cervix of irregular morphology with the disrupted cervical stroma ring (long arrow). The thickened distal part of the rectal and anal canal wall (hollow arrow). Multiple round low signal myometrial lesions – uterine fibroids (short arrows).

Histological images of the cervical and anal canal lesions are shown in Figure 3. Microscopic examination revealed, in the cervical specimen, moderately differentiated invasive squamous cell carcinoma (SCC) (Fig. 3A), featuring a strong and diffuse immunohistochemical p16 reaction in the neoplastic cells (Fig. 3B). In the search of HPV high risk, the polymerase chain reaction (PCR) reaction was performed, which confirmed the presence of high risk HPV (HPV 16 and additional one from the group of High Risk HPV 31,33,35,39,51,52,56,58,59,66,68) in the cervical SCC. The microscopic examination of the biopsy specimen from the anal canal lesion revealed moderately differentiated adenocarcinoma (Fig. 3C) with patchy p16 immunohistochemical stain in the cancer cells (Fig. 3D), while the polypectomy specimen showed the conventional serrated adenoma with low grade dysplasia. The low grade dysplasia was also found at the diathermied margin. The PCR reaction excluded the presence of high risk HPV in the anal canal adenocarcinoma, followed by the negative result for low risk HPV PCR test. In addition real-time PCR was carried out for anal canal adenocarcinoma confirming NRAS mutation and excluding KRAS and BRAF mutation. Physical, endoscopic and histological examinations were supplemented with magnetic resonance imaging (MRI). Examination revealed prolapsed cervix of irregular morphology with the disrupted cervical stroma ring (long arrow). Multiple low T2 signal round myometrial lesions was suggestive of uterine fibroids (short arrows; hollow arrows). MRI also revealed irregular mass-like thickening of the distal part of the rectum and anal canal, superiorly to the anal sphincters. Both internal and external sphincters seemed uninvolved. The lesion was confined to the intestine wall and did not infiltrate ischioanal fat nor adjacent pelvic structures (Fig. 4).

**Figure 5:**
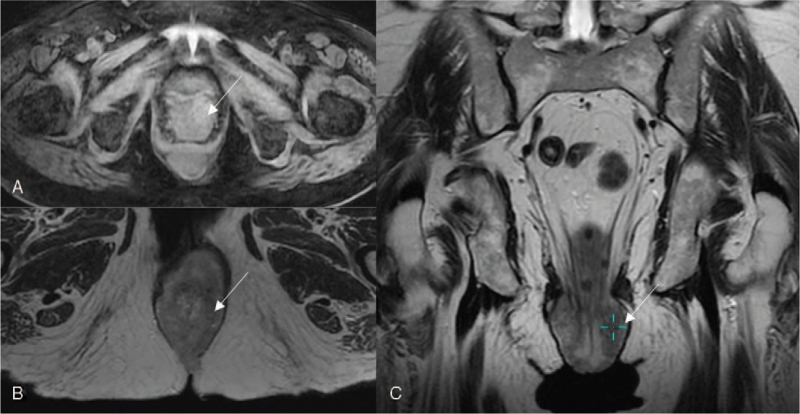
Axial LAVA image after intravenous contrast administration (A) and T2-weighted: axial (B) and coronal (C) images. A lesion measuring 40 × 20 × 25 mm was identified in the cervix, hyper intense in LAVA sequence (A) and of low signal on T2-weighted images (B, C). There is no evidence of vaginal mucosa, bladder and rectal invasion.

The next lesion measuring 40 × 20 × 25 mm was identified in the cervix. It demonstrated a low signal on the T2-weighted sequence and a high signal in LAVA after contrast administration. No evidence of vaginal mucosa, bladder and rectal invasion was found. Superficial parametral invasion on the left was suspected (Fig. 5).

**Figure 6:**
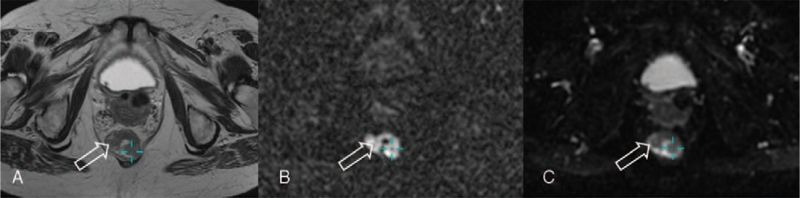
Axial T2-weighted (A), diffusion-weighted (DWI) b = 1000 s/mm^2^ (B) and apparent diffusion coefficient (ADC) map (C) magnetic resonance images. Diffusion-weighted magnetic resonance images suggested the malignant character of the thickened (A) rectal and anal canal wall. High signal on B-value diffusion-weighted images (B) with corresponding low signal on ADC maps (C) confirmed water diffusion restriction – a sign of malignancy (hollow arrows).

Diffusion-weighted magnetic resonance sequences suggested the malignant character of the thickening (A) of the rectal and anal canal wall. High signal on B-value diffusion-weighted MRI (B) with corresponding low signal on apparent diffusion coefficient (ADC) map (C) confirmed water diffusion restriction – a sign of malignancy (hollow arrows; Fig. 6). T stage corresponded to the size of the primary tumour assessed by measuring in its longest diameter on T2-weighted MR images. In our case, the maximum tumour diameter was 23 mm.

Based on the above data, cervical G2 squamous cell carcinoma FIGO II A 2 was diagnosed in the prolapsed cervix with the coexisting G2 anal canal adenocarcinoma pT1N0M0.

**Figure 7:**
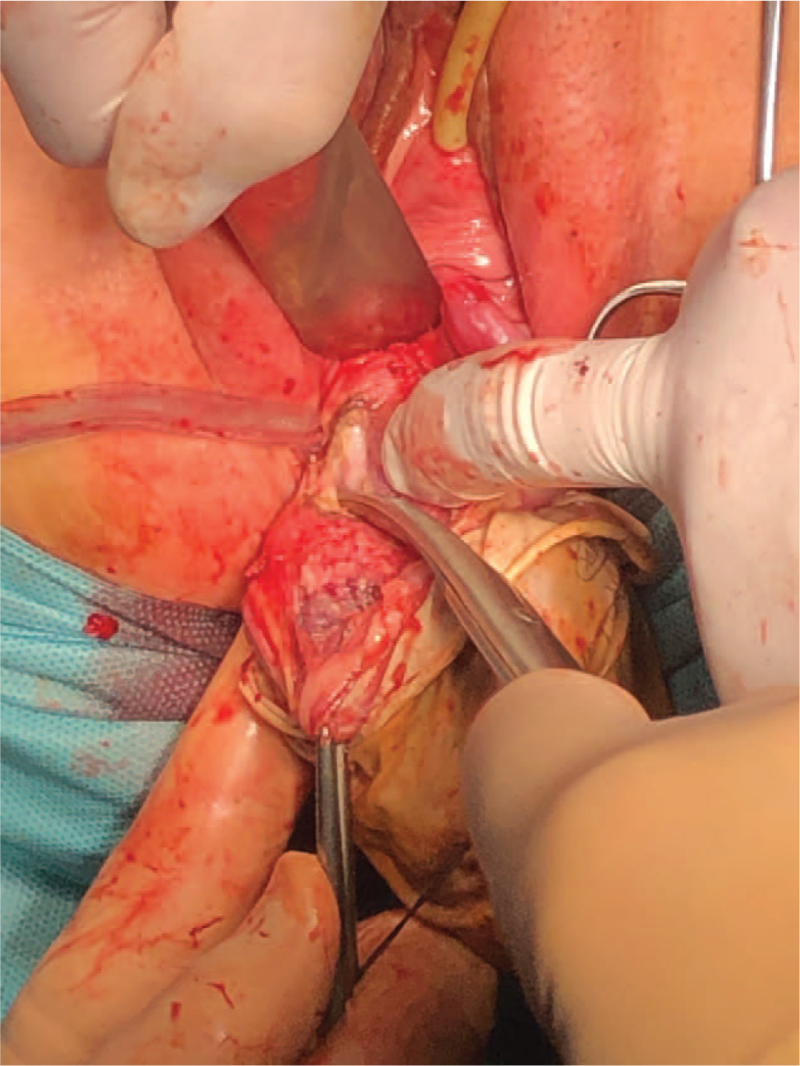
Intraoperative view of vaginal radical hysterectomy with the visible knee of the right ureter.

Due to the predominance of symptoms related to CC and prolapse, the patient was offered a treatment consisting of, at the first stage, radical vaginal hysterectomy with extensive colpectomy preceded by laparoscopic pelvic and paraaortic lymphadenectomy, followed by the removal of the anus in the second stage. The patient agreed to some of the proposed treatment, however, she did not consent to the anal extirpation. Therefore, only laparoscopic pelvic and paraaortic lymphadenectomy with radical vaginal hysterectomy were performed. The key step of vaginal radical hysterectomy with the isolation of the right ureter is shown in Figure 7. The dissected ureteric angulation, known as knee of the ureter, allowed excision of the parametria.

The postoperative course was uneventful. The patient was discharged on day 5. Microscopic examination of postoperative specimens revealed the cervix containing partially exophytic tumor measuring 2.5 × 3.5 × 7.5 cm, which appeared to be G2 HPV-associated squamous cell carcinoma, focally invading beyond the cervix into surrounding adipose tissue, sparing the parametria (pT2a2), with clear peripheral margins. The perineural and vascular space involvement was identified. Out of 13 regional lymph nodes examined, 2 showed metastasis (2/13).

Due to the refusal of both anal extirpation and adjuvant chemotherapy, after a multidisciplinary consultation, the patient was qualified for radiotherapy (RT). She was transferred to the Radiotherapy Department at the Regional Hospital in Tarnow. The RT was dedicated as definitive treatment for ACC and adjuvant therapy for CC. RT was delivered via a two-step 3D conformal technique. The lymph nodes affected by SCACC and regional lymph nodes of anal cancer were included in step 1 and the dose of 50.4 Gy in 28 fractions was delivered. In step 2, boost of 3.6 Gy in 2 fractions to the primary anal canal was added. As the patient did not want to undergo resection of the anus, we planned brachytherapy in the third stage, as a way of dose escalation. The prescribed dose was defined as 98% of the planning target volume that should receive 98% of the dose. organ and risks were contoured, including the peritoneal space (bowel bag), bladder and bilateral femoral head. All constrains of tolerance doses for organ and risks were maintained. The treatment was tolerated well, however, Common Terminology Criteria for Adverse Events (v4.0), including grade 2 diarrhoea and anal canal colitis, were demonstrated. RT was completed without discontinuation through treatment.

**Figure 8:**
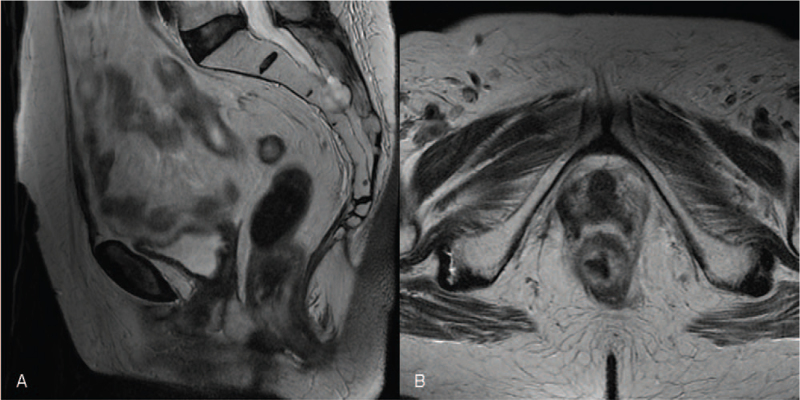
Sagittal (A) and axial (B) T2-weighted magnetic resonance images performed 45 days after treatment. No signs of previous-seen mass-like thickening of the rectal and anal canal wall on T2-weighted images.

**Figure 9:**
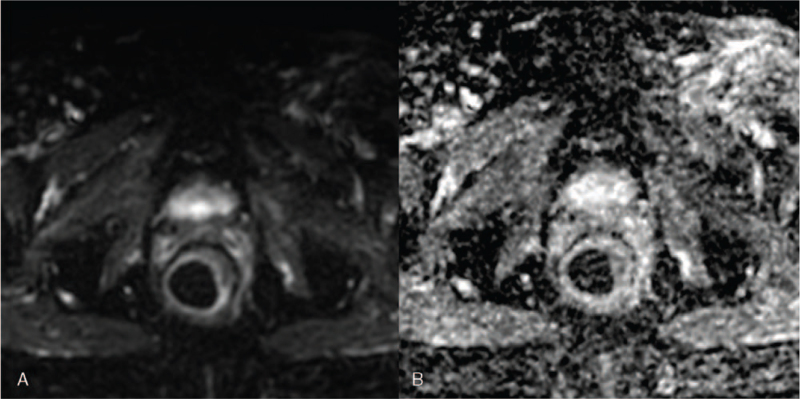
Diffusion-weighted magnetic resonance images performed 45 days after treatment. b = 1000 s/mm^2^ (A) and apparent diffusion coefficient map (B). Complete response: no evidence of suspected regions of water diffusion restriction.

MRI performed on 45 postoperative days after radical vaginal hysterectomy and RT demonstrated a complete response. No sign of previous-seen mass-like thickening of the rectal wall on T2-weighted images (Fig. 8). Diffusion-weighted imaging (Fig. 9) confirmed no evidence of suspected regions of water diffusion restriction.

The duration of follow-up was 6 months.

Informed written consent was obtained from the patient for publication of this case report and accompanying images.

## 3 Discussion

To the best of the authors’ knowledge, no report on synchronous CC with prolapse and ACC is available in the literature. Only 1 study was found dealing with the abovementioned 2 synchronous neoplasms but without pelvic organ prolapse.^[1]^ It was worth noting, the case reported in this study was contrary to the one described by us, since the more advanced of synchronous tumours was ACC.

Coexistence of CC with pelvic organ prolapse is rare. It is estimated that 1% of CC patients develop uterine prolapse.^[6]^ On the other hand, the incidence of CC among patients with uterine prolapse is 0.14% to 1%.^[10–12]^

The incidence of ACC is very low reaching 1.5:100,000 but rising, due to spreading of HPV and HIV, autoimmune disorders and cigarette smoking.^[13]^

Synchronous neoplasms may have a common etiological factors, HPV infection being a well recognized one. A good example is HPV-associated synchronous neoplasms of the uterine cervix and the throat.^[14]^ In the presented case, HPV HR was identified only in 1 neoplasm – cervical SCC, not in the anal adenocarcinoma. Meanwhile, Saleem et al^[15]^ observed that women with HPV infection have a higher SIR of anal cancer. Several other studies have found HPV infection as an etiological agent of ACC.^[16–19]^

Uterine prolapse may have an ambiguous impact on the aetiology of CC. CC occurs in the elderly women, in whom a prolapsed cervix is exposed to injuries that predispose to the process of carcinogenesis.^[4]^ On the one hand, it is associated with the lack of contact with vaginal secretions and such a “protective” role against carcinogenesis is suggested by some of the authors.

The presence of CC was evident and only needed to be confirmed with a biopsy. The diagnosis of cancer of the anal canal was more challenging because the patient described herein did not report any symptoms. This was due to the fact that in our case we were dealing with the early stage of this tumour. According to Durot et al,^[13]^ more advanced cases are characterized by pain, hardening, ulceration, bleeding, fistulas, and weight loss.

The diagnosis of ACC was based on the obligatory per rectum examination to assess the CC stage and on the rectoscopic image with histopathology. Additionally, the diagnosis was confirmed with MRI. In our case, this examination allowed for an accurate diagnosis, staging and follow-up. According to Ferrer Marquez et al,^[20]^ other useful diagnostic methods may be rectal endoscopic ultrasound, CT of the chest and abdominal cavity for the assessment of metastases and positron emission tomography. MRI will also determine the clinical advancement of CC. It should be noted that imaging methods, including MRI, were not approved by FIGO until the 2018 classification.^[21]^

It is important to distinguish synchronous neoplasms from metastatic ones because synchronous neoplasms have a better prognosis and a different regimen treatment.^[22]^ In our case, the evidence of synchronous nonmetastatic character of the tumours was based on different histological forms of the neoplasms: squamous cell carcinoma and adenocarcinoma. According to Song et al,^[22]^ in the case of uncertain histology, further molecular tests (eg, microsatellite instability, lack of chromosomal heterozygosity, and inactivation of cloned X chromosomes; or screening for mutation sin female genital tract tumor genes such as *PTEN*, *TP53*, *KRAS*, and *CTNNB1*) and cytogenetic analyses (eg, IHC of b-catenin) are often required. In our case, the diagnosis of synchronous cancers based on clinical findings and histopathology was obvious.

Available literature lacks uniform guidelines regarding the treatment of synchronous cases of cervical and anal cancer. However, some general guidelines concerning the treatment of synchronous neoplasms are available in the literature. One of them suggests the second cancer to be treated in the shortest possible time.^[11]^ In the case presented herein, the choice of the cancer that was treated first was conditioned by the presence of bothersome symptoms, that is, bleeding.

According to the current literature, a second primary tumor should be resected as early as possible, and its treatment should be similar to that of a single tumor.^[22]^

In our case, the patient refused to undergo surgical treatment of the second tumour, that is, the cancer of the anal canal. In line with Song et al^[22]^ recommendations, we took into account the tumour stage, the patient's age and life expectancy, and decided to apply nonoperative methods.

## 4 Conclusion

Most synchronous neoplasms do not have generally accepted management algorithms. First of all, it results from lack of wide prospective randomized trials. Additionally, it was due to different patient characteristics, comorbidities, patients’ therapeutic decisions, and the different stages of coexisting tumours.

In the presented case, we have the coexistence of 2 rare cancers of the cervix with prolapse and cancer of the anal canal. An additional factor was the patient's refusal to consent to the removal of the anus. Due to the lack of treatment algorithms, the presented case may serve as an example of individualized treatment of neoplasms found in this study and an urgent call for robust study on personalized treatment in all synchronous tumours.

## Author contributions

**Conceptualization:** Andrzej Skręt.

**Data curation:** Andrzej Skręt, Joanna Trawińska, Joanna Bielatowicz, Mariusz Książek, Andrzej Radkowski, Jaromir Kargol, Bogusław Gawlik.

**Formal analysis:** Andrzej Skręt, Joanna Bielatowicz, Mariusz Książek, Beata Niewęgłowska- Guzik.

**Funding acquisition:** Bogusław Gawlik.

**Project administration:** Joanna Skręt- Magierło.

**Supervision:** Joanna Trawińska.

**Visualization:** Andrzej Skręt.

**Writing – original draft:** Andrzej Skręt, Joanna Trawińska, Mariusz Książek, Andrzej Radkowski, Jaromir Kargol.

**Writing – review & editing:** Andrzej Skręt, Joanna Trawińska, Joanna Bielatowicz, Mariusz Książek, Edyta Barnaś.
